# Characterisation of IL-23 receptor antagonists and disease relevant mutants using fluorescent probes

**DOI:** 10.1038/s41467-023-38541-2

**Published:** 2023-05-19

**Authors:** Charles S. Lay, Albert Isidro-Llobet, Laura E. Kilpatrick, Peter D. Craggs, Stephen J. Hill

**Affiliations:** 1 Division of Physiology, Pharmacology and Neuroscience, School of Life Sciences, University of Nottingham, Nottingham, NG7 2UH UK; 2 Centre of Membrane Proteins and Receptors, University of Birmingham and Nottingham, The Midlands, UK; 3 Chemical Biology, Medicine Design, GlaxoSmithKline, Stevenage, SG1 2NY UK; 4 Division of Bimolecular Science and Medicinal Chemistry, School of Pharmacy, Biodiscovery Institute, University of Nottingham, Nottingham, NG7 2RD UK; 5 Crick-GSK Biomedical Linklabs, Medicine Design, GlaxoSmithKline, Stevenage, SG1 2NY UK

**Keywords:** Receptor pharmacology, Receptor pharmacology, Proteins, Biological fluorescence

## Abstract

Association of single nucleotide polymorphisms in the IL-23 receptor with several auto-inflammatory diseases, led to the heterodimeric receptor and its cytokine-ligand IL-23, becoming important drug targets. Successful antibody-based therapies directed against the cytokine have been licenced and a class of small peptide antagonists of the receptor have entered clinical trials. These peptide antagonists may offer therapeutic advantages over existing anti-IL-23 therapies, but little is known about their molecular pharmacology. In this study, we use a fluorescent version of IL-23 to characterise antagonists of the full-length receptor expressed by living cells using a NanoBRET competition assay. We then develop a cyclic peptide fluorescent probe, specific to the IL23p19:IL23R interface and use this molecule to characterise further receptor antagonists. Finally, we use the assays to study the immunocompromising C115Y IL23R mutation, demonstrating that the mechanism of action is a disruption of the binding epitope for IL23p19.

## Abstract (web summary)

The single nucleotide polymorphism C115Y in the IL-23 receptor is associated with autoinflammatory diseases. Here the authors demonstrate that this mutation prevents the binding of a fluorescent cyclic peptide and IL-23 to the IL-23 receptor.

## Introduction

Class I and Class II Cytokine receptors are single transmembrane domain (TMD) containing proteins that associate to form homo- or heteromeric receptor complexes that bind cytokine ligands^1^. Typically, homo or hetero-dimeric complexes of these receptor subunits engage a monomeric or dimeric protein ligand with binding leading to the transphosphorylation and activation of JAK proteins constitutively associated to the intracellular domains (ICD) of the receptor subunits. Activation of JAK proteins in turn leads to phosphorylation of the ICD and the recruitment of signal transducers^2^. Despite many cytokines being important drug targets, for example Interleukin-4 (IL-4), IL-5 and IL-6^3–5^, there has been limited development of peptide or small molecule receptor antagonists and the receptor pharmacology of this target class remains under explored^6^. Instead, the development of biologic cytokine antagonists and small molecule signalling inhibitors has been pursued^7^. Whilst biologic therapies have been highly effective, they are expensive to produce, do not cross the blood brain barrier and are usually confined to systemic administration through sub-cutaneous injection^8^. Small molecule inhibition of cytokine signal transducers, for example JAK proteins, suffers from numerous side effects due to their use in a variety of pathways^9^. The successful development of small molecule peptide antagonists of cytokine receptors would enable specific inhibition of individual cytokine pathways and could allow local administration for example through a topical or gut restricted treatment for diseases such as psoriasis or Crohn’s disease respectively.

IL-23 is a heterodimeric cytokine from the IL-12 family made up of a disulphide linked complex of the proteins IL12p40 and IL23p19^10^. The cytokine binds to a heterodimeric receptor made up of the components IL12Rβ1 and IL23R^11^, with IL12p40 specifically engaging IL12Rβ1 and IL23p19 binding to IL23R^12,13^. Ligand binding induces JAK2 associated to the ICD of IL23R and TYK2 bound to the ICD of IL12Rβ1 to trans-phosphorylate and activate each other, enabling the phosphorylation of the IL23R ICD and further signal transducers such as STAT3^14^. IL-23 signalling is involved in initiating pro-inflammatory immune responses, with IL23R loss of function mutants leading to increased infection with mycobacterial pathogens^15,16^, however the pathway has also been shown to be instrumental in the development of several auto-inflammatory diseases^17^. Biologic treatments are available that antagonise either the IL23p19 or IL12p40 subunits of the IL-23 cytokine and cyclic peptide antagonists of the receptor are in clinical trials^18^.

Despite the reports of several classes of peptide IL23R antagonists including linear and cyclic peptides with competitive and non-competitive binding mechanisms^19–22^, no probes have yet been outlined and validated to characterise this nascent drug class’s interaction with the receptor. Instead, these peptides have been validated using either, functional read-outs which do not allow detailed pharmacological characterisation of the peptide’s binding mechanism or immobilised purified protein techniques such as ELISA and SPR which do not allow the measurement of the interactions with the full-length dimeric receptor expressed in the cell membrane^19–23^. This second point is important as it has previously been demonstrated that when the subunits of the IL-23 receptor are purified as truncates their affinity for IL-23 is markedly reduced^24,25^.

Fluorescently labelled probes are key tools for discovering and characterising receptor antagonists^26–28^. The interaction of G Protein Coupled Receptors (GPCRs) and their ligands have been extensively characterised through this approach, using techniques such as Fluorescence Polarisation (FP), Förster Resonance Energy Transfer (FRET) or Bioluminescence Resonance Energy Transfer (BRET) to construct competition assays^29^. These assays facilitate screens to identify binders and enable the characterisation and development of drug-like molecules^30,31^. In addition, fluorescent probes can be used to label targets and are especially useful when antibodies specific for a receptor are difficult to create^32^.

In this study we validate the use of a previously reported TAMRA labelled version of IL-23 (IL23-TMR^24^) for use in the characterisation of reported receptor antagonists at both IL23R and IL12Rβ1 and design and characterise a fluorescent cyclic peptide probe (P630-TMR) specific to the IL23R receptor subunit only. We then use P630-TMR to measure IL23R specific inhibitors, demonstrating how the probes can be used together to classify IL-23 receptor antagonists. Finally, we use the fluorescent probes and other proximity-based techniques to define the mechanism of action of the immunocompromising C115Y mutation^15^ by demonstrating that the mutation disrupts the IL23R:IL23p19 interface.

## Results

### Characterising reported IL-23 receptor antagonists using IL23-TMR

**Fig. 1:**
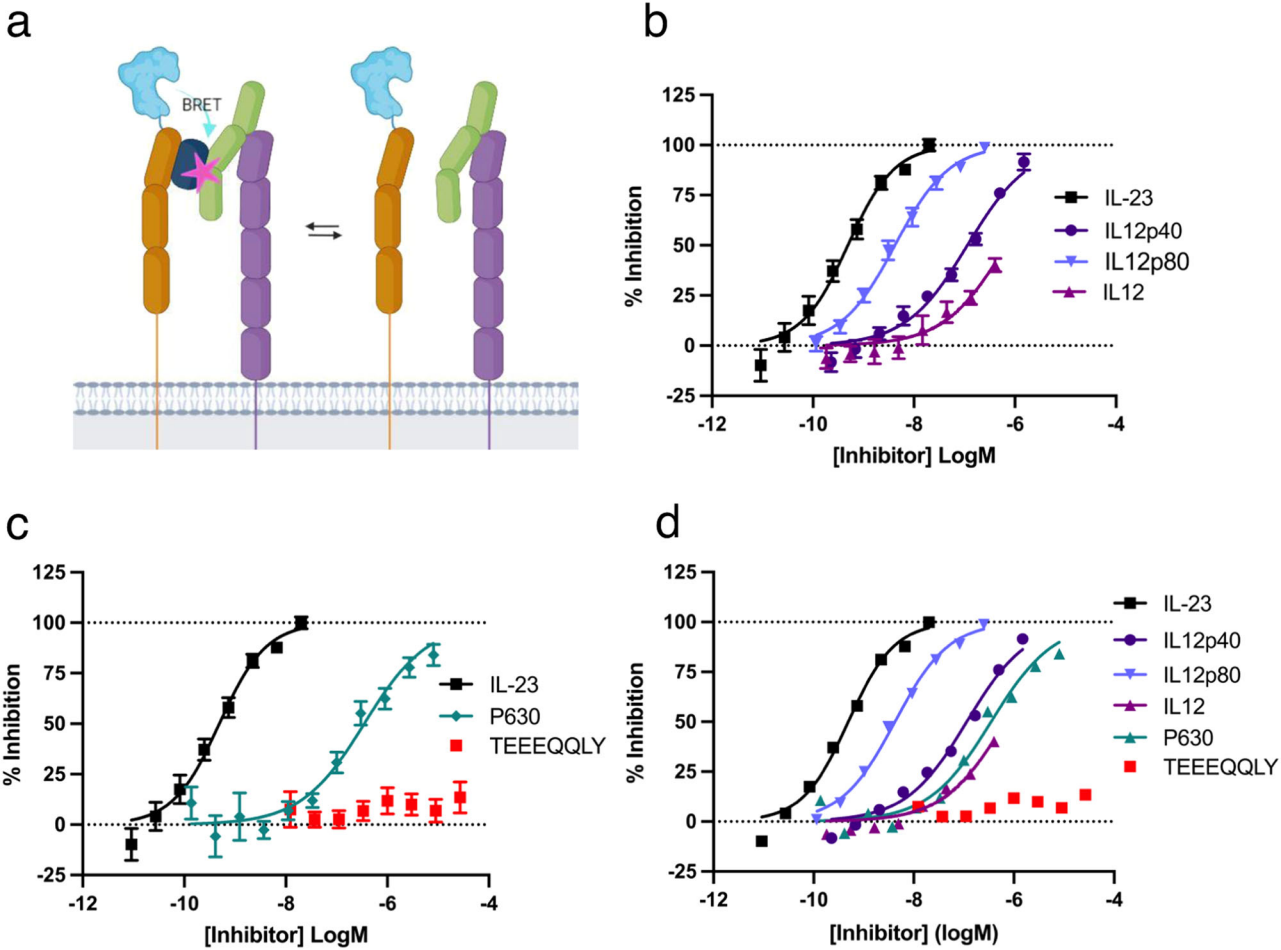
Using IL23-TMR to characterise IL-23 receptor antagonists. **a** A schematic demonstrated the principles of the BRET competition assay using IL12p40 as an example. Schematic created with Biorender.com. IL23R is shown in orange, IL12Rβ1 in purple, IL23p19 in blue, IL12p40 in green and the presence of a TAMRA fluorophore by a pink star. The competitive inhibition of the binding of 300 pM IL23-TMR by increasing concentrations of (**b**) endogenously produced IL-12p40 containing proteins and (**c**) synthetic IL-23 receptor antagonists with previously generated IL-23 competition data^24^ for comparison. **d** The combined data from **b** and **c** plotted without error bars. In **b** and **c**, data are mean ± SEM from 5 independent experiments conducted in either duplicate or triplicate. Details on absolute number of data points included for each concentration are provided in the associated Source Data file. K_i_ values obtained in the 5 independent experiments are shown in Table 2.

We have previously shown that IL23-TMR can be competitively displaced by unlabelled IL-23^24^. To assess if IL23-TMR can be displaced by further reported IL-23 receptor antagonists we tested several antagonists using this IL23-TMR competition assay on HEK293T cells transiently expressing NL-IL23R and IL12Rβ1 (Fig. 1). The antagonism of several endogenously secreted proteins that contain the IL-23 subunit IL12p40 were tested (Fig. 1b, d). IL12p40 itself displaced IL23-TMR with a *K*_i_ of 12.4 ± 1.4 nM (*n* = 5; 392-fold weaker than IL-23). IL12p80, a homodimer of IL12p40, inhibited IL-23 binding with a *K*_i_ of 469 ± 210 pM (*n* = 5; 17.4-fold weaker than IL-23). IL-12, a heterodimeric cytokine consisting of IL-12p40 and IL-12p35, exhibited a low level of inhibition that did not fully displace IL23-TMR over the concentration range tested. Next the IL23-TMR displacement of reported synthetic IL-23 receptor antagonists was tested (Fig. 1c, d). An octapeptide with the sequence TEEEQQLY that was reported to be a negative allosteric modulator (NAM) of the IL-23 receptor^23^ was found not to displace IL23-TMR. However, a cyclic peptide known as Peptide 630 (P630) reported in a patent filing describing oral IL23R antagonists^19^ displaced IL23-TMR with a *K*_i_ of 21.8 ± 3.4 nM (*n* = 5; 690-fold weaker than IL-23).

### Synthesis and characterisation of fluoro-labelled P630

**Fig. 2:**
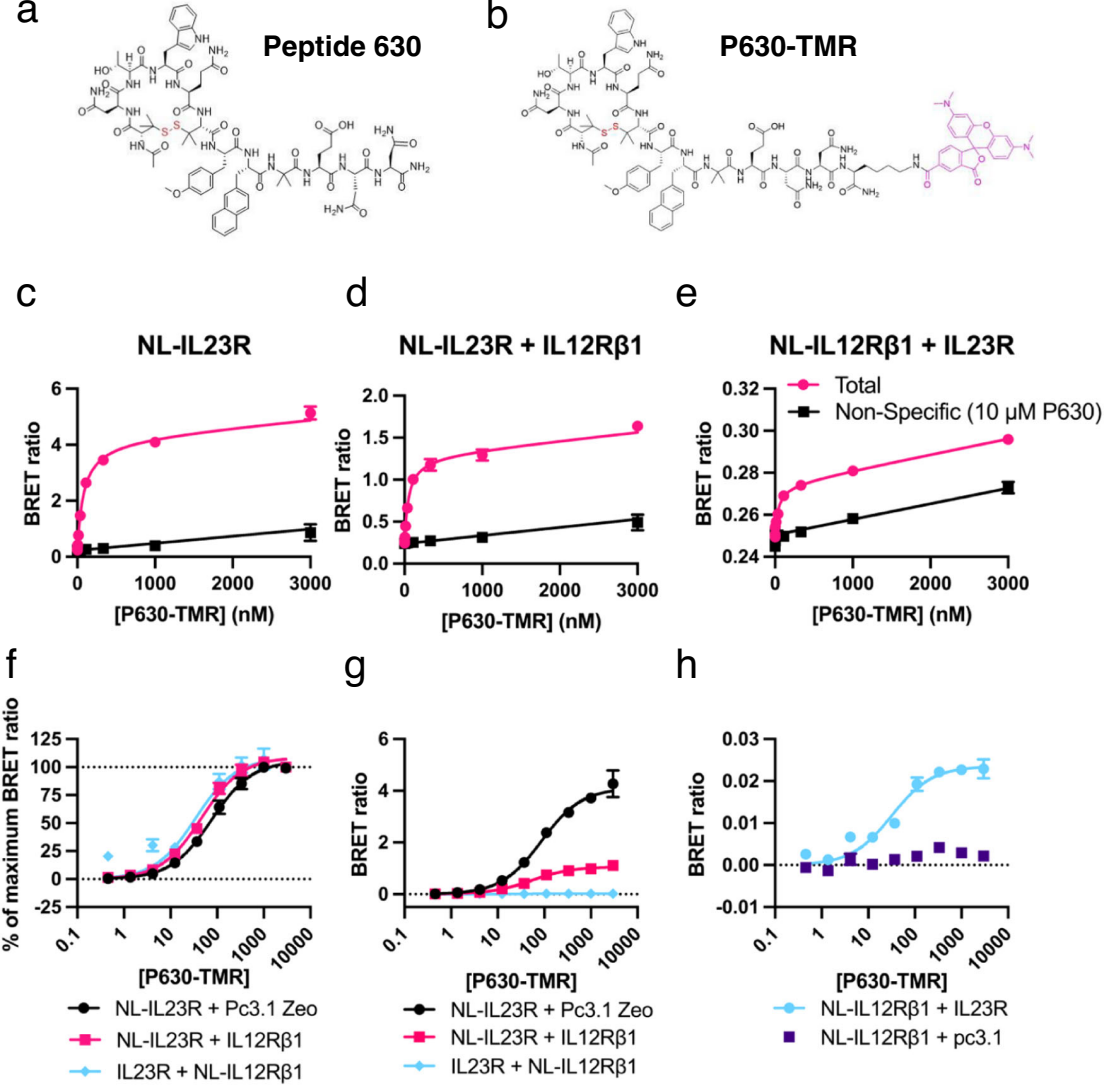
Creation and characterisation of a fluorescent cyclic peptide probe for IL23R. The chemical structures of (**a**) P630 and (**b**) P630-TMR. The BRET ratio measured when increasing concentrations of P630-TMR were applied to cells expressing (**c**) NL-IL23R alone, (**d**) NL-IL23R with IL12Rβ1 or (**e**) NL- IL12Rβ1 and IL23R in the presence or absence of 10 μM P630. **f** The data shown in **c**–**e** normalised to % maximum BRET ratio. **g** The BRET data shown in **c**–**e** with non-specific binding subtracted. **h** The specific binding of P630-TMR to cells expressing NL- IL12Rβ1 in the presence or absence of IL23R. Data are normally mean ± SEM from five independent experiments. Details on absolute number of data points included for each concentration are provided in the associated Source Data file. In each individual saturation experiment triplicate determinations were normally made for total binding and duplicate measurements were made for non-specific binding. K_d_ values obtained in the 5 independent experiments are shown in Table 2.

With the aim of creating a small molecule fluorescent probe for IL23R we designed and synthesised a TAMRA labelled version of P630. The resulting labelled probe P630-TMR, consisted of P630 conjugated to TAMRA via a lysine residue bound to the *C-*terminus of the peptide (distal to the cyclised portion of the molecule; Fig. 2b; Table 1). To test whether we could measure the binding of P630-TMR to the receptor via BRET, increasing concentrations of the probe was applied to cells expressing NL-IL23R with IL12Rβ1, NL-IL23R alone and NL- IL12Rβ1 with IL23R (Fig. 2c–e). It was observed that P630-TMR bound specifically with comparable affinity (Mean ± SEM, *n* = 5; *K*_d_ = 51.1 ± 7.3, 101 ± 4 and 55.2 ± 10.5 nM respectively) to cells expressing each of these combinations of constructs (Fig. 2c–e; Table 2). Whilst P630-TMR bound each of the receptor combinations with similar affinities, the maximum binding (Bmax) BRET ratio was different depending on the constructs expressed (Fig. 2f) with the highest Bmax measured for the interaction with NL-IL23R expressed in isolation (4.04-fold higher than NL-IL23R with IL12Rβ1), and the lowest Bmax measured for the interaction with cells expressing NL- IL12Rβ1 with IL23R (42.53-fold lower than NL-IL23R with IL12Rβ1). A standard curve was used to predict the expression level of NanoLuc labelled receptor in each experiment (Supplementary Fig. 1).

**Table 1:** LS-MS/MS profiles for labelled (TMR) and unlabelled P630

Peptide	Mass/charge (m/z)	Charge (z)	Mono-isotopic mass (M + H)^a^	Retention time (min)	Purity (%)
**P630-TMR**	1103.96	2	2206.92	10.53	>95
**P630**	833.30	2	1665.60	9.92	>95

^a^Mono-isotopic mass at a single charge assuming the peptide takes up a single proton. Mono-isotopic mass [M + H] is calculated as (m/z)*z-(z-1). The difference in mono-isotopic mass between P630-TMR and P630 is 541.32 which is equivalent to the theoretic mass of 541.45 for the addition of 5-carboxytetramethylrhodamine and lysine to the C-terminus of P630.

**Table 2:** The affinities of antagonists and labelled probes described in this study

Probe	Assay	Constructs expressed	K_i_ or K_d_	*N*
IL12p40	IL23-TMR Competition (Fig. 1)	NL-IL23R and IL12Rβ1	12.4 ± 1.4 nM	5
IL12p40	P630-TMR Competition (Fig. 5)	NL-IL23R and IL12Rβ1	Did not displace labelled probe	5
TEEEQQLY	IL23-TMR Competition (Fig. 1)	NL-IL23R and IL12Rβ1	Did not displace labelled probe	5
TEEEQQLY	P630-TMR Competition (Fig. 5)	NL-IL23R and IL12Rβ1	Did not displace labelled probe	3
P630	IL23-TMR Competition (Fig. 1)	NL-IL23R and IL12Rβ1	21.8 ± 3.4 nM	5
P630	P630-TMR Competition (Fig. 5)	NL-IL23R and IL12Rβ1	6.32 ± 1.06 nM	5
IL12p80	IL23-TMR Competition (Fig. 1)	NL-IL23R and IL12Rβ1	469 ± 210 pM	5
IL12p80	P630-TMR Competition (Fig. 5)	NL-IL23R and IL12Rβ1	Did not displace labelled probe	5
IL-23	IL23-TMR Competition (Fig. 1)	NL-IL23R and IL12Rβ1	31.6 ± 7.7 pM (Lay et al.^24^)	13
IL-23	P630-TMR Competition (Fig. 5)	NL-IL23R and IL12Rβ1	22.6 ± 5.2 pM	5
P630-TMR	Binding assay (Figs. 3 and 4)	NL-IL23R and IL12Rβ1	51.1 ± 7.3 nM	5
P630-TMR	Binding assay (Figs. 3 and 4)	NL-IL23R	101 ± 4 nM	5
P630-TMR	Binding assay (Figs. 3 and 4)	NL-IL12Rβ1 and IL23R	55.2 ± 10.5 nM	5
P630-TMR	Binding assay (Figs. 3 and 4)	NL-IL12Rβ1	Did not bind	3
P630-TMR	Binding assay (Figs. 3 and 4)	HiBit-IL23R and LgBit- IL12Rβ1	53.5 ± 4.4 nM	3

Values are mean ± SEM obtained from *N* separate experiments.

**Fig. 3:**
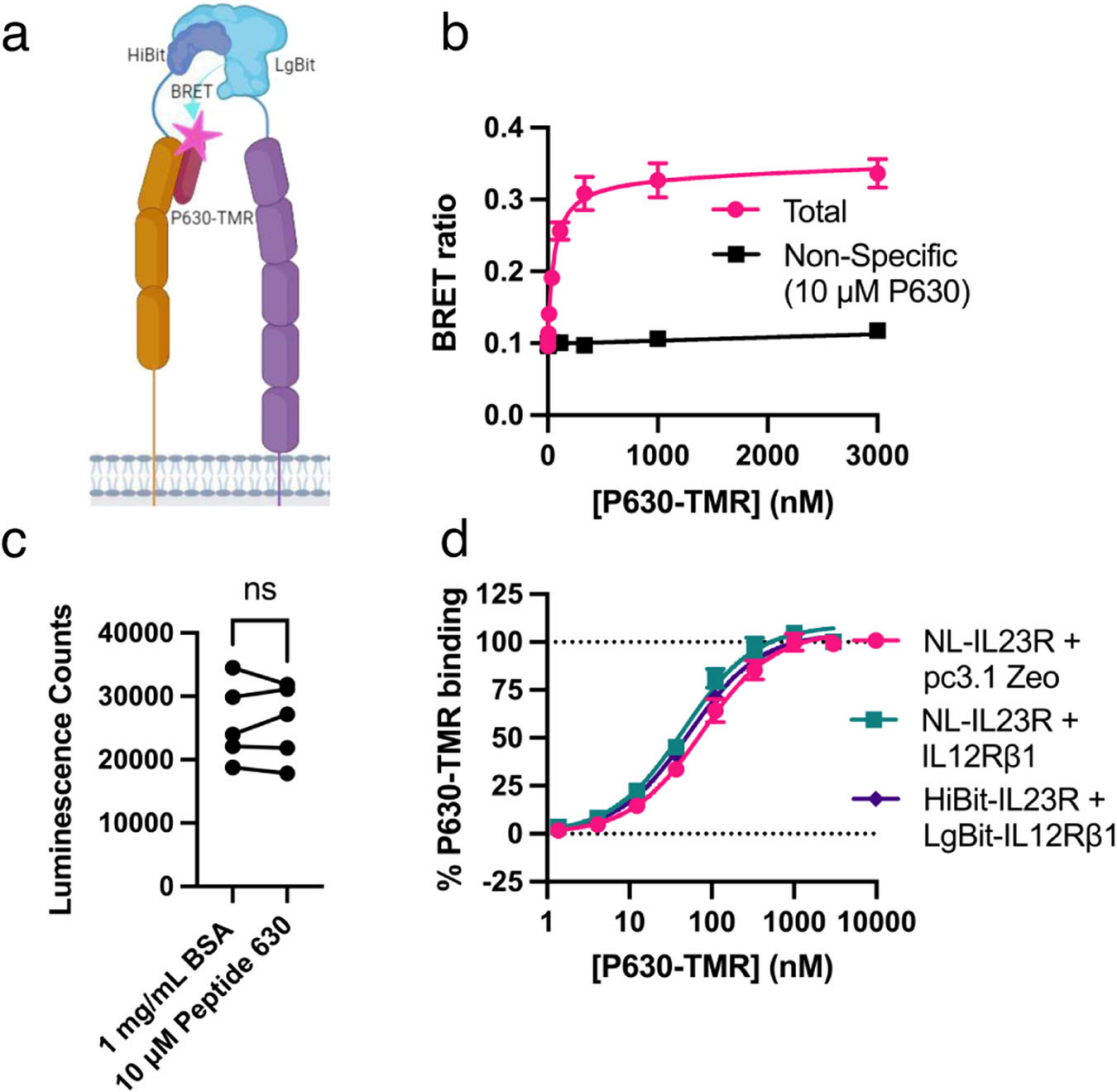
P630-TMR binding to binary NanoBiT IL-23 receptor complexes. **a** A schematic depicting the NanoBiT assay methodology, with IL23R shown in orange and IL12Rβ1 shown in purple. Schematic created with Biorender.com. **b** The BRET ratio generated when increasing concentrations of P630-TMR were applied to cells expressing NanoBiT complemented receptors. **c** The effect of P630 on NanoLuc complementation and hence receptor dimerisation. **d** The data from (**b**) normalised to % of P630-TMR specific Bmax and plotted with data generated in Fig. 2d, e for reference. Data in **b** are mean ± SEM from three independent experiments. In each individual experiment, triplicate determinations were made for total binding and duplicate or triplicate determinations made for non-specific binding. Data in **c** are the mean values obtained in five independent experiments, each conducted with six replicates. Ns no significant difference (two-tailed, paired *t* test of the paired mean values obtained in each experiment; *p* = >0.05). Data in **d** are the mean ± SEM from five (NL-IL23R and NL-23R + IL12Rβ1) or three (HiBit-IL23R + LgBit-IL12Rβ1) independent experiments. Source data are provided as a Source Data file.

The P630 peptide was originally designed through a phage display study using IL23R alone^19^ and another peptide from the patent has been mapped to the IL23R:IL-23 interface^33^, therefore P630-TMR is likely to bind specifically to the IL23R subunit. To test this hypothesis, we measured the binding affinity of P630-TMR to cells expressing NL-IL12Rβ1 in isolation (Fig. 2g) and found that in the absence of IL23R expression there was no specific binding. Finally, we utilised a NanoBiT BRET system in which a complemented luciferase with fragments bound to both receptor subunits (Fig. 3) can be used as a BRET donor to measure interactions with binary receptor complexes^34^. P630-TMR specifically bound to these binary complexes with a similar affinity (*K*_d_ = 53.5 ± 4.4 nM; mean ± SEM, *n* = 3) to that measured for the previously measured tagged receptor combinations (Fig. 3b–c; Table 2). The effect of unlabelled P630 on NanoLuc complementation between HiBit-IL23R and LgBit-IL12Rβ1 was also tested (Fig. 3c), with the results demonstrating that P630 did not affect receptor dimerisation (two-tailed, paired *t* test; *p* = >0.05).

### Competition experiments utilising P630-TMR

**Fig. 4:**
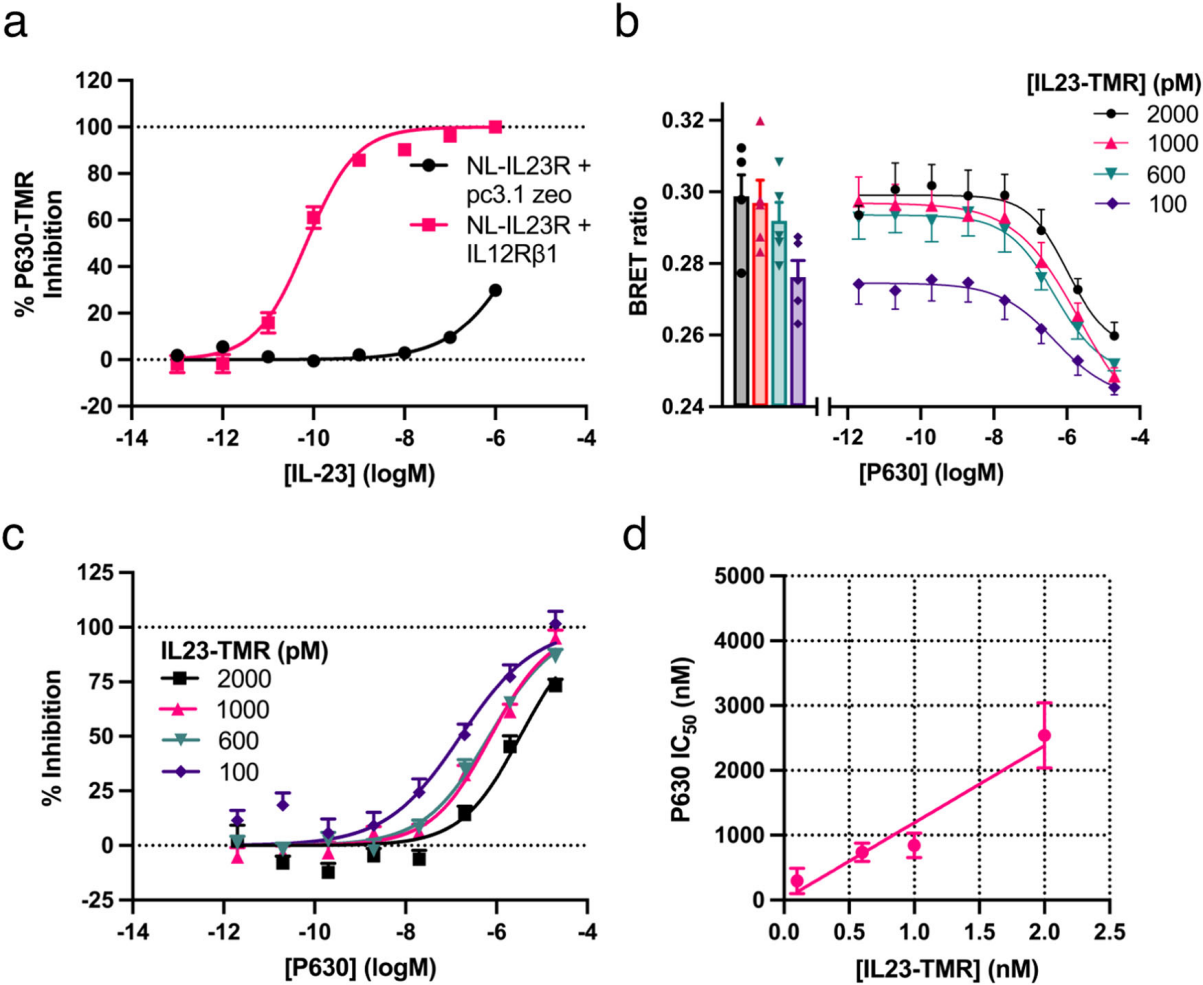
The displacement of P630-TMR by IL-23 is dependent on the co-expression of IL12Rβ1 with IL23R. **a** The displacement of 75 nM P630-TMR by increasing concentrations of IL-23 from cells expressing NL-IL23R in the presence or absence of IL12Rβ1. **b** The displacement of different concentrations of IL23-TMR by increasing concentrations of P630. **c** The data shown in **b** normalised to % inhibition (total inhibition was defined by an excess of IL-23). **d** The relationship between P630 IC_50_ determined in **c** and the concentration of IL23-TMR used. Data are mean ± SEM from four (**a**: NL-IL23R + pc3.1 zeo) or five independent experiments performed in triplicate. In **b** the bars show the mean values obtained in each of five independent experiments and the data points show the mean ± SEM of the values obtained in the five independent experiments. Source data are provided as a Source Data file.

To test if P630-TMR displacement could be used to measure the affinity of unlabelled IL-23, a competition assay was undertaken in which P630-TMR was displaced by increasing concentrations of IL-23 from cells expressing either NL-IL23R in isolation or NL-IL23R co-expressed with IL12Rβ1. It was observed that IL-23 displaced P630 with a much higher *K*_i_ when both subunits of the receptor were expressed (Fig. 4a). When NL-IL23R was expressed alone P630-TMR was not fully displaced over the concentration of IL-23 tested. When NL-IL23R was co-expressed with IL12Rβ1 a *K*_i_ of 22.6 ± 5.2 pM (mean ± SEM, *n* = 5) was measured for IL-23 which was similar to the value measured previously using the displacement of IL23-TMR (31.6 ± 7.7 pM^24^).

To assess if the interaction of P630 with IL-23 was competitive, multiple concentrations of IL23-TMR were displaced by P630. Consistent with a competitive interaction increasing concentrations of IL23-TMR shifted the measured P630 IC_50_ to higher levels (Fig. 4b). The mean value generated for P630 *K*_i_ was 38.0 ± 9.0 nM ( ± SEM, *n* = 25). The Cheng-Prusoff equation was then used as previously described^24^ to calculate the *K*_d_ value of IL23-TMR from the linear regression of the data plotted in Fig. 4D and the measured P630 *K*_i_ value. A rearrangement of the Cheng-Prusoff equation yields the relationship:

1 $${{IC}}_{50}={K}_{i}+\frac{{K}_{i}}{{K}_{d}}x[L]$$

This allowed the K_d_ of IL23-TMR to be determined (32.0 pM) from the slope of the line in Fig. 4d. The experiment was also repeated with different concentrations of P630-TMR being competed by IL-23. Interestingly over the concentration range of P630-TMR tested there was not a significant change in the measured IL-23 IC_50_ (Supplementary Fig. 2).

**Fig. 5:**
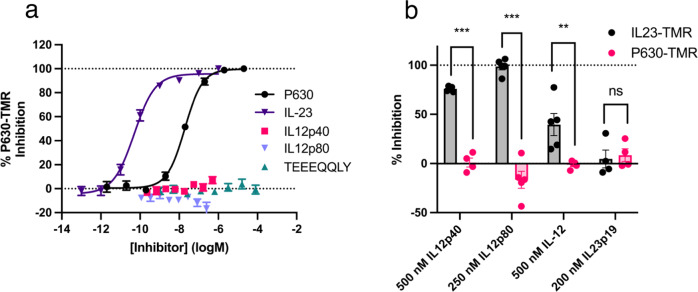
P630-TMR measures antagonism at the IL23R/IL23p19 interface. **a** The displacement of 75 nM P630-TMR by increasing concentrations of various IL-23 receptor binders. **b** The displacement of either 75 nM P630-TMR or 300 pM IL23-TMR by a single concentration of several antagonists. Data in **a** are mean ± SEM from three (TEEEQQLY), four (P630, IL12p40) or five (IL-23, IL12p80) independent experiments performed in triplicate. Data in **b** are mean ± SEM from five independent experiments performed in triplicate apart from 200 nM IL23p19 (4 experiments) and 500 nM IL12p40 with P630-TMR (4 experiments). In **b** statistical analysis (two-tailed, non-paired, *t*-test) was performed on the mean values obtained in each independent experiment. ** indicates a statistical significance of *p* < 0.01. *** indicates a statistical significance of *p* < 0.001. Absolute *p* values in **b** were 0.000001 (IL-12p40), 0.000002 (IL12p80), 0.0069 (IL12) and 0.74 (IL23p19). Source data are provided as a Source Data file.

We then tested if P630-TMR could be used to characterise other reported IL-23 receptor antagonists from cells expressing NL-IL23R and IL12Rβ1 (Fig. 5a). As expected, unlabelled P630 was able to displace P630-TMR yielding a *K*_i_ of 6.32 ± 1.06 nM which was similar to the value measured using IL23-TMR (21.8 ± 3.4 nM). TEEEQQLY, IL12p80 and IL12p40 did not displace P630-TMR. Given that IL12p40 displaced IL23-TMR but not P630-TMR we tested single concentrations of an IL23p19-Fc fusion protein and IL-12 (Fig. 5b). A 200 nM concentration of IL23p19-Fc exhibited a very low level of inhibition of both IL23-TMR and P630-TMR. IL-12 gave no inhibition of P630-TMR binding at a concentration that partially displaced IL23-TMR.

### Cytokine and peptide binding to a disease linked IL23R mutant

**Fig. 6:**
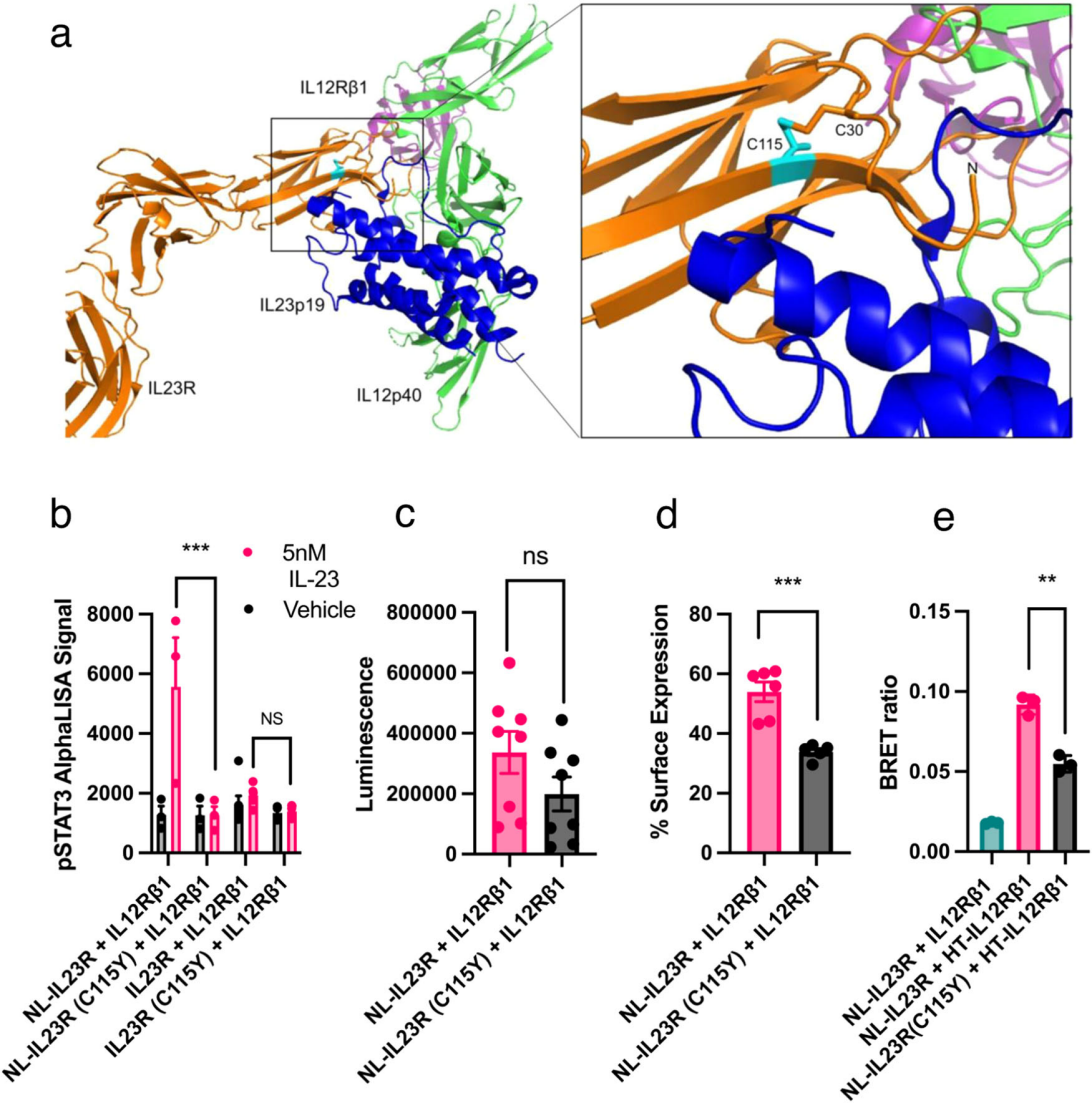
A single nucleotide polymorphism linked to immunodeficiency blocks binding of IL23-TMR and P630-TMR. **a** Left: Crystal structure of *N*-terminal domains 1–3 of IL23R (orange) in complex with IL-23 (IL23p19 = blue, IL12p40 = green) and the *N*-terminal domain of IL12Rβ1 (PDB: 6WDQ^13^). Right: The interface between IL23R and IL23p19 with C115 coloured cyan. **b** The phospho-STAT3 signal generated when cells expressing different constructs were treated with 5 nM IL-23. Data are mean ± SEM from five or six (untagged) or three (NanoLuc tagged) independent experiments conducted in triplicate. For the IL23R + IL12Rβ1 data set, one outlier (11108.7) was detected using the Grubbs test (with alpha = 0.001) and removed from the statistical analysis. Statistical significance of the C115Y mutation on the alphaLISA data was assessed using a 2-way ANOVA with Tukey’s multiple comparison test. ***p < 0.001. The *p* value for the comparison of NL tagged construct response was 0.0001 and the *p* value for the comparison of untagged receptor was 0.951. **c** Luminescence signal measured when IL12Rβ1 was expressed with either NL-IL23R or the C115Y mutant. Data are mean ± SEM generated from the mean luminescence values of seven (C115Y) or eight independent experiments performed in pentuplicate. **d** The proportion of luminescence from **c** that emanated from extracellular protein (as defined by the reduction in luminescence after treatment with 60 μM NanoLuc extracellular inhibitor). Data are mean ± SEM generated from the mean luminescence values of five (C115Y) or six independent experiments performed in triplicate. **e** The BRET ratio generated from HaloTag-618 labelled cells expressing NL tagged wildtype or C115Y IL23R with HT-IL12Rβ1 or unlabelled IL12Rβ1. Data are mean ± SEM generated from the mean BRET values of three independent experiments performed in pentuplicate. Statistical significance of differences in mean values shown in **c**–**e** were measured using a 2-sided, non-paired *t* test with ** indicating a *p* value of <0.005 and *** indicating a *p* value of <0.0005. The *p* values for the results shown in **c**, **d** and **e** were *p* = 0.148, 0.0005 and 0.0013 respectively. Source data are provided as a Source Data file.

A single nucleotide polymorphism resulting in a cysteine to tyrosine substitution in the *N-*terminal domain of IL23R was previously reported in patients demonstrating a deficient immune response to Mycobacterial pathogens, with this mutation shown to block IL-23 induced STAT3 phosphorylation^15^. Due to the position of the C115Y mutation at the IL23R:IL23p19 interface (Fig. 6a) and the residue’s role in tethering a ‘hook-like’ projection of IL23R that interacts with IL23p19^25^, we hypothesised that the loss of function of the C115Y mutant could be due to impaired binding of IL-23. To test this, we created untagged and NanoLuc tagged versions of IL23R with the C115Y mutation. We found that, as previously reported^15^, the mutation blocked STAT3 phosphorylation (Fig. 6b). Using the NanoLuc tag as a marker of expression it was demonstrated that the C115Y mutation could be successfully surface expressed in HEK293T cells, although the mutation resulted in a 41% reduction in total NL-IL23R and a 37% reduction in the proportion of NL-IL23R on the cell surface as compared to wildtype (Fig. 6c, d). Using an intra-receptor NanoBRET assay to measure the basal association of receptor subunits (as previously described in Lay et al.^24^), we discovered that the C155Y mutant still associated with a fluorescently labelled HaloTag (HT) tagged IL12Rβ1 construct although this was reduced by 50.1% from the level of association measured for wildtype IL23R, a reduction consistent with the protein’s reduced total and surface expression (Fig. 6e).

**Fig. 7:**
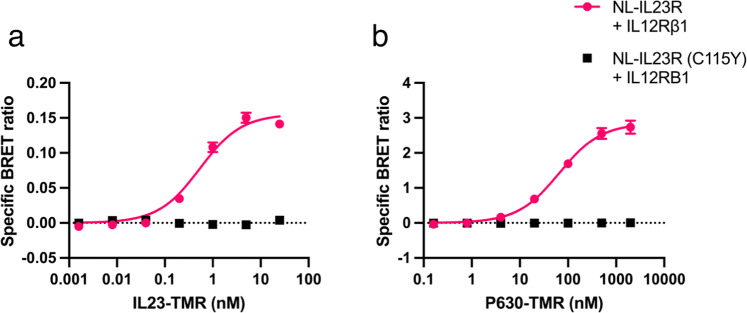
Effect of C115Y on the binding of IL23-TMR and P630-TMR. BRET ratio generated when increasing concentrations of (**a**) IL23-TMR or (**b**) P630-TMR were applied to cells expressing wildtype (pink) or C115Y (black) mutant receptor. Data are mean ± SEM from five (C115Y) or four (wildtype) independent experiments conducted in duplicate or triplicate. Details on absolute number of data points included for each concentration are provided in the associated Source Data file.

Despite the association of the mutant NL-IL23R with HT-IL12Rβ1, when these receptor complexes were treated with IL-23, we did not observe the characteristic decrease in BRET that has been previously associated with a ligand induced conformational change in the position of the *N-*terminal domains (Supplementary Fig. 3^24^). We then tested if the mutant IL23R could bind IL23-TMR, finding that whilst a specific BRET signal was measured from control cells expressing the wildtype receptor, IL23-TMR did not bind to C115Y mutated IL23R (Fig. 7a). To test if P630-TMR and IL23-TMR shared a common binding epitope in the *N*-terminal region of IL23R we then measured the binding of P630-TMR to cells expressing wildtype and mutant receptor, finding that the C115Y mutation also blocked specific binding of P630-TMR (Fig. 7b).

## Discussion

The availability of validated chemical probes is a vital resource for the interrogation of emerging drug targets. Whilst there are few reports of small molecule antagonists of cytokine receptors, a growing list of cyclic peptide inhibitors of the IL-23 receptor indicates that the target may be tractable for antagonists beyond traditional antibody-based drugs^20–23,35^. In this study we used a fluorescently tagged version of IL-23 to measure antagonism of the IL-23 receptor by IL12p40, an endogenous protein antagonist^36^ and P630, a peptide from an emerging class of cyclic peptide IL23R inhibitors^19^. We then developed P630 into a fluorescent probe for the IL-23 receptor termed P630-TMR, through conjugation with the fluorophore TAMRA.

By measuring the binding of P630-TMR to cells expressing the cytokine receptor subunits in isolation and together, using NanoBRET ligand binding assays, we determined that P630-TMR bound specifically to IL23R. We then established that the mechanism of action of the IL23R C115Y loss of function mutation, is a disruption of the IL23R:IL23p19 binding epitope and used this result to demonstrate that the IL23R binding epitope for P630-TMR and IL23-TMR is most likely conserved, as both probes cannot bind this mutant.

We observed that whilst P630-TMR bound with comparable affinity to cells expressing NL-IL23R in isolation or with IL12Rβ1, the BRET efficiency was much higher in the absence of IL12Rβ1. We have previously shown that the IL-23 receptor subunits associate in the absence of ligand with the *N-*termini in proximity^24^. A possible explanation for the observed modulation in BRET efficiency with IL12Rβ1 is a modification of the position of the IL23R conjugated NanoLuc-tag caused by the association of the subunits in an inactive complex. The further reduction in BRET efficiency observed when the NanoLuc tag is placed on the *N*-terminus of IL12Rβ1 is most likely due to the increased distance between the *N*-terminus of IL12Rβ1 and the P630-TMR binding epitope, however the successful measurement of a BRET signal is consistent with the receptor subunits forming a complex in the absence of IL-23, as has been previously suggested^24,37^.

Fluoroprobes are useful tools for drug discovery because they can be used to identify the competitive binding of molecules that share a common binding site. We found that we could displace P630-TMR with IL-23, unlabelled P630 and to a low degree with an IL23p19-Fc fusion protein and so it is likely P630-TMR could be used to characterise binders of the IL23p19/IL23R interface. Furthermore, by positioning the NanoLuc tag on the *N-*terminus of IL12Rβ1, displacement of P630-TMR could be measured from unlabelled IL23R. This would be advantageous as structural studies have demonstrated that the *N*-terminal domain of IL23R is closely involved in ligand binding. Using this approach, the interaction of unmodified IL23R binders with untagged full-length IL23R could be measured in the membranes of living cells, enabling a more accurate recapitulation of in vivo drug-target interactions.

We were able to demonstrate that the interaction between IL23-TMR and P630 was competitive, reinforcing the hypothesis that P630 binds to a shared epitope with IL-23. However, the lack of a conventional competitive interaction when we displaced P630-TMR with IL-23 indicates that observations of the interaction at the IL23R interface may be biased by the presence of the additional IL-23 binding site on IL12Rβ1, which acts as an additional allosteric site from which P630 cannot displace IL-23.

IL-23 engages its receptor in a bivalent interaction in which IL23R specifically engages IL23p19 and IL12Rβ1 specifically engages IL12p40^12,13^. This means that potential competitive antagonists could be directed to either the IL12Rβ1 or IL23R epitopes, however due to the promiscuous pairing of IL12Rβ1 with IL12Rβ2 to form the IL-12 receptor complex, all IL23R antagonists reported thus far have been directed at the IL23R interface, making them specific to IL-23 signalling. Using fluoro-labelled IL-23 we were able to measure antagonism at both the IL23R and IL12Rβ1 interfaces. As shown by the displacement of IL23-TMR but not P630-TMR by IL12p40, a protein that is likely to bind to IL12Rβ1 as a monomer as it has been shown to bind to the same protein when part of both the IL-12 and IL-23 complexes^13,38^. We were also able to displace IL23-TMR but not P630-TMR with IL-12, presumably through competition at the IL12p40:IL12Rβ1 interface. This finding may represent an endogenous mechanism through which IL-23 signalling is regulated by the presence of IL-12, through competition for IL12Rβ1. IL12p80, a homodimer of IL12p40 reported to act as an antagonist of IL-12 signalling and to have its own functional responses, also inhibited IL23-TMR binding. This is presumably through the IL12p40:IL12Rβ1 interface as P630-TMR was not displaced by an equivalent concentration of IL12p80. Another interesting observation was the increased inhibition of IL23-TMR by IL12p80 when compared to IL12p40 (26.4-fold). Although IL12p80 contains two IL12p40 molecules this does not account for the increase in competition. Taken together with previous reports of murine IL12p80’s increased inhibition of IL-12 binding compared to IL12p40^39^, this observation indicates that homodimerization of IL12p40 increases the molecule’s affinity for IL12Rβ1, for example through the bivalent engagement of IL12Rβ1 homodimers.

We also tested the competitive displacement of an octapeptide that is a reported allosteric antagonist of the IL-23 receptor, finding that it did not displace IL23-TMR or P630-TMR. As the inhibition of functional response by this molecule was not tested in our study, we cannot comment on its efficacy as an IL-23 receptor antagonist, however the results indicate that, as reported, the peptide does not competitively bind to either of the IL-23 binding epitopes^23^.

A disadvantage of using a fluoroprobe competition assay in drug discovery, is that allosteric interactions may not be identified if they do not displace the probe used. A good example in our study is the lack of displacement of P630-TMR by IL12p40. The advantageous nature of the approach described here, is that by screening potential antagonists through both P630-TMR and IL23-TMR competition assays, ‘hits’ can be further sorted into IL23p19 competitive (if they displace P630-TMR) or IL12p40 competitive or allosteric (if they displace IL23-TMR). If an allosteric molecule binds to the receptor, preventing activation by IL-23 but not impacting IL23-TMR binding, this could be identified by one of the many functional assays that have been described for the pathway^40,41^. Currently drug discovery efforts targeting the IL-23 receptor would either need to screen molecules against purified protein in a biochemical assay, an approach which does not accurately represent the activity of the receptor constituents on the cell surface^24^, or screen ‘hits’ found in functional assays for activity against signal transducers that would result in non-specific inhibition of the pathway.

This study describes the use of P630-TMR as a competitive probe for IL23R antagonists, however this is not the only application for a membrane receptor specific fluorescent probe. Further applications of P630-TMR could be the fluorescent labelling of cells for imaging or flow cytometry experiments^32^. The approach outlined in this study to measure competitive interactions at IL23R should enable better characterisation of an emerging class of peptide inhibitors. As the tractability of further cytokine receptors for peptide or small molecule antagonism is established (for example^42,43^) this technology could be applied in a similar way to further under-characterised drug targets. In addition, the identification of the mechanism of action of the C115Y IL23R mutation could have implications for patients who have immunodeficiencies caused by this variant. Our results indicate that whilst the mutation reduces surface expression, development of a chaperone treatment would be unlikely to restore function due to the additional disruption of IL-23 binding caused by the mutation.

## Methods

### Mammalian expression constructs

NL-IL23R, NL-IL12Rβ1, IL12Rβ1 and pc3.1 zeocin plasmid expression vectors were prepared as previously described^24^. IL23R in a pc3.1 zeocin vector was custom synthesised at Genscript. HiBit-IL23R and LgBit-IL12Rβ1 expression vectors were created by restriction cloning IL12Rβ1 or IL23R into pc3.1 zeo expression vectors with *N-*terminal HiBit or LgBit fusion (kindly donated by Dr Mark Soave^44^). The C115Y IL23R mutant was generated from NL-IL23R and IL23R plasmids using a Phusion site directed mutagenesis kit (ThermoFisher) according to the manufacturers’ instrutions. The oligonucleotide primers used (Merck) were:

Forward primer 5’-CAAGAGACACTGATATaTGGAAAAGACATTTC-3’

Reverse primer 5’-AAAATGTTTGGGACATTCAGCAG-3’

The lower case a in the forward primer shows the mutation from G to A to covert cysteine to tyrosine. PCR products were then digested with Dpn1 for 15 min at 37 °C to digest methylated (template) DNA. The mutated C115Y IL23 sequence was then excised from the PCR generated plasmid vector and cloned back into the original template vector. Sequences were confirmed using Sanger sequencing (DEEPSEQ, University of Nottingham).

### Cell culture and transfection

Human Embryonic Kidney 293 T (HEK293T) cells (ATCC # CRL-3216) were cultured using Dulbecco’s Modified Eagle Medium (DMEM) (Merck) with 10% Fetal Bovine Serum (FBS) (Gibco) at 37.5 °C with 5% CO_2_. These cells were tested for Mycoplasma infection using a MycoStrip TM 100 kit (Invivogen) and found to be negative. Transient transfections for experiments outlined in Fig. 6 were conducted by seeding 100 μL of 2 × 10^5^ cells/ml concentrated HEK293T cells into poly-D-lysine (PDL) coated white 96 well clear bottom assay microplates (Greiner) and incubating overnight. The following day transfection mixes were made up that contained a 4:1 ratio of IL23R to IL12Rβ1 constructs to a total concentration of 100 ng per well with a 0.3 μL per well of FuGENE HD transfection reagent (Promega) in OptiMEM (Gibco). These mixtures were incubated for 10 min before being added to the plate at 5 μL per well. The cells were then incubated overnight to allow expression. For all other experiments 2 mL containing 4 × 10^5^ cells were seeded into 6 well plates and incubated 5 h before being transiently transfected with 100 μL per well of a transfection mix containing 2 μg of DNA and 6 μL of FuGENE HD. These cells were then incubated overnight before being re-suspended the following day and seeded into PDL coated white assay microplates at 100 μL containing 3 × 10^4^ transfected cells. Cells were then incubated overnight before the experiment. Estimates of total expression levels for the different NL-constructs were made by comparing the luminescence levels achieved to that of a NanoLuciferase standard curve measured under the same conditions (Supplementary Fig. 1).

### Peptide antagonists and P630-TMR

Recombinant HEK293T produced IL23p19-Fc (Sinobiological; Cat #: 13062-H02H) and IL-12 (Sinobiological; Cat #: CT050-HNAH), CHO produced IL12p40 (Peprotech; Cat #: 200-12p40), and BTI-Tn-5B1-4 produced IL12p80 (Merck; Cat #: SRP3075) were purchased from commercial suppliers as lyophilised solids which were re-constituted in PBS (Merck) with 1 mg/ml BSA (Merck). TEEEQQLY, P630 and P630-TMR were custom synthesised by Cambridge Research Biochemicals. TEEEQQLY was reconstituted in PBS with 1 mg/ml BSA P630 and P630-TMR were reconstituted as 1 mM stocks with 10% DMSO in PBS. P630 and P630TMR were analysed by liquid chromatography-tandem mass spectroscopy (Supplementary Figs. 4–8) by Cambridge Research Biochemicals. HPLC was performed with an ACE3 C18-300 300 Å column (150 × 2.1 mm) at a flow rate of 0.35 ml/min. The buffer used was 0.1% trifluoracetic acid in water with a gradient increasing from 2–70% of 0.1% trifluoracetic acid in acetonitrile over 13 min. Absorbance was measured at 230 nm. Mass spectrometry was performed using Maldi-TOF. The difference in mono-isotopic mass between P630-TMR and P630 was 541.32 which is equivalent to the theoretic mass of 541.45 for the addition of 5-carboxytetramethylrhodamine and lysine to the C-terminus of P630 (Table 1).

### NanoBRET ligand binding and competition experiments

Media was removed from white 96-well microplates containing transfected cells and replaced with fluoroprobe in the presence or absence of antagonist in 50 μL Hanks Balanced Salt Solution (HBSS) with 1 mg/mL BSA (Merck). After a 1-hour incubation at 37 °C without CO_2,_ 5 μL of HBSS containing 77 μM furimizine was added to each well (final concentration 7 μM), the plate incubated for 5-minutes and the BRET signal measured using a PheraStar FS plate reader (BMG Labtech; data acquisition software version 5.41; MARS analysis software version 3.32) with a 450 ± 30 nm and >550 nm long pass filters. P630-TMR binding assays were normalised to a final DMSO concentration of 0.1%.

### Phospho-STAT3 signal transduction assays

Phosphorylation of STAT3 was measured using an AlphaLISA Surefire Ultra assay as previously described^24^. Briefly, transfected HEK293T cells were plated into a poly-D-lysine coated 96 well tissue culture microplate and then incubated overnight. The following day media was replaced with serum free DMEM and the cells incubated for 3 h. Media was then replaced with HBSS containing 1 mg/ml BSA and increasing concentrations of IL-23 and the cells incubated for 30 min. An AlphaLISA SureFire Ultra assay kit (Perkin Elmer #ALSU-PST3) was then used to measure STAT3 phosphorylation at residue Tyr705. Assays were performed according to the manufacturers’ instructions.

### Total and surface expression quantification

Total receptor expression was quantified by measuring the luminescence from transfected cells treated with 7.7 μM furimizine in 50 μL HBSS for 10 min using a PheraStar FS plate reader. To quantify what proportion of this luminescence was a result of surface expressed protein, half of these cells were supplemented with 60 μM NanoLuc extracellular inhibitor (Promega) during the 10-minute incubation prior to the measurement of luminescence.

### Intra-receptor NanoBRET experiments

Transfected cells had media replaced with DMEM with 10% FBS containing 100 nM HaloTag 618 ligand (Promega) and were incubated for 5 h at 37 °C with 5% CO_2_. Media was then replaced with HBSS with or without 5 nM IL-23 and the cells were incubated for a further hour in the absence of CO_2_. Following this 5 μL of HBSS with 77 μM furimizine was added to each well and the BRET signal was measured on a PheraStar FS plate reader using 460 ± 80 nm and >610 long pass filters.

### Data analysis

BRET ratio values were generated by dividing the acceptor signal by the donor signal values. Data analysis was carried out using GraphPad Prism (GraphPad Software version 9.4.1).

Ligand binding data were fitted with the equation:

2 $$B{RET\; ratio}=\frac{{B}_{\max }[A]}{([A]+{K}_{D})}+(\left(B\left[A\right]\right)+C).$$

Where ‘Bmax’ is the maximum specific binding BRET signal, ‘[A]’ is fluorescent probe concentration, ‘B’ is the non-specific binding component’s slope and ‘C’ is the Y intercept.

Specific binding data was generated by subtracting the BRET values gained in the presence of an excess of unlabelled probe from the BRET ratios gained in the absence of unlabelled probe. Specific binding data were fit using the equation:

3 $$Y=\frac{{B}_{\max }[B]}{({\left[B\right]+K}_{D})}$$

where ‘B_max_’ is the maximum specific binding signal of the curve and ‘[B]’ is the concentration of fluorescent probe.

Antagonist competition data were fit with the equation:

4 $$Y={B}_{\min }+\frac{{B}_{\max }-{B}_{\min }}{1+{10}^{({LogXC}50-[A])C}}$$

Where ‘B_max_’ is the maximum BRET signal, ‘B_min_’ is the minimum BRET signal, ‘LogXC50’ is the log of the 50% inhibiting concentration of antagonist, ‘[A]’ is the concentration of antagonist and ‘C’ is the Hill slope.

Statistical analyses were carried out in GraphPad Prism. Statistical analysis was carried out on different populations of cells which had undergone separate treatments.

The crystal structure of *N*-terminal domains 1–3 of IL23R in complex with IL-23 and the *N*-terminal domain of IL12Rβ1 (PDB: 6WDQ) was visualised using PyMOL Molecular Graphics System, Version 2.0 Schrödinger, LLC.

### Reporting summary

Further information on research design is available in the Nature Portfolio Reporting Summary linked to this article.

## Supplementary information

Supplementary Information

Reporting Summary

## Data Availability

The data that support this study are available from the corresponding authors upon request. Source data are provided with this paper.

## Source data

Source Data
