# Supplementary material for: Characterisation of IL-23 receptor antagonists and disease relevant mutants using fluorescent probes
Source: Nat Commun. 2023 May 19;14:2882. doi: 10.1038/s41467-023-38541-2 (PMC10199020; doi:10.1038/s41467-023-38541-2)
Supplement: Supplementary file 1 — Supplementary Information [file 41467_2023_38541_MOESM1_ESM.pdf]

## Supplementary Figures.

# Characterisation of IL-23 receptor antagonists and disease relevant mutants using fluorescent probes

Charles S. Lay[1,2,3], Albert Isidro-Llobet[3], Laura E Kilpatrick[2,4], Peter D. Craggs[3,5,*] and Stephen J. Hill[1,2,*]

[1] Division of Physiology, Pharmacology and Neuroscience, School of Life Sciences, University of Nottingham, Nottingham NG7 2UH, UK

[2] Centre of Membrane Proteins and Receptors, University of Birmingham and Nottingham, The Midlands, UK

[3] Chemical Biology, Medicine Design, GlaxoSmithKline, Stevenage SG1 2NY, UK

[4] Division of Bimolecular Science and Medicinal Chemistry, School of Pharmacy, Biodiscovery Institute, University of Nottingham, NG7 2RD, UK

[5] Crick-GSK Biomedical Linklabs, Medicine Design, GlaxoSmithKline, Stevenage SG1 2NY, UK

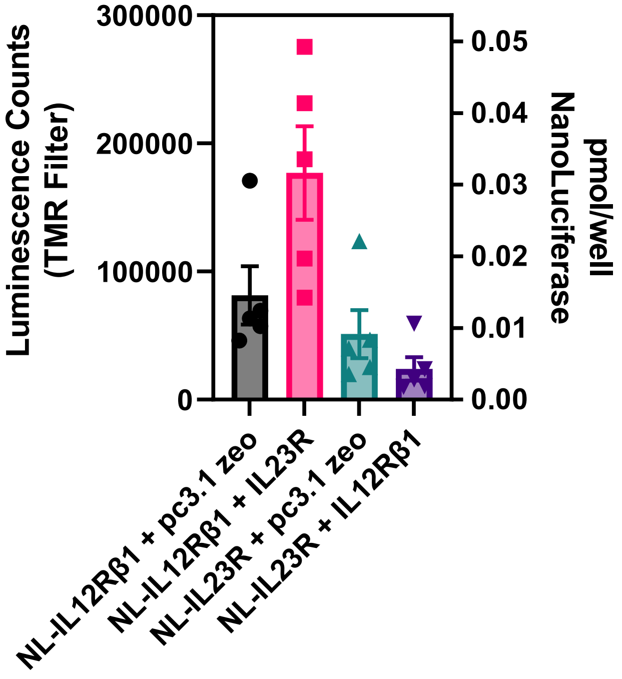


**Supplementary Fig. 1: Prediction of receptor expression label using a standard curve**. A previously generated standard curve of purified NanoLuc [24] was used to predict the expression level of IL-23 receptor components from luminescence values (in the absence of ligand) measured in the binding experiments shown in Figure 2. Data are mean ± SEM from five independent experiments performed in triplicate.

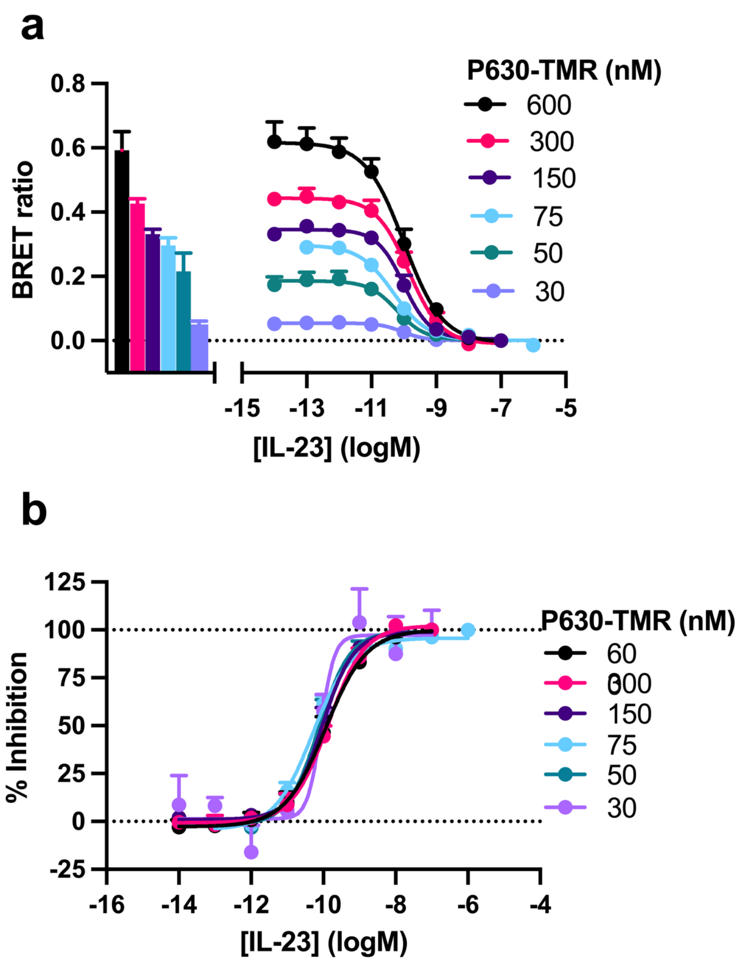


**Supplementary Fig. 2: Non-competitive interaction between IL-23 and P630-TMR.** (a) Competitive displacement of several concentrations of P630-TMR by increasing concentrations of IL-23. (b) The data from (a) normalised to % of the maximum inhibition of the BRET signal. Data are mean ± SEM from five independent experiments performed in triplicate.

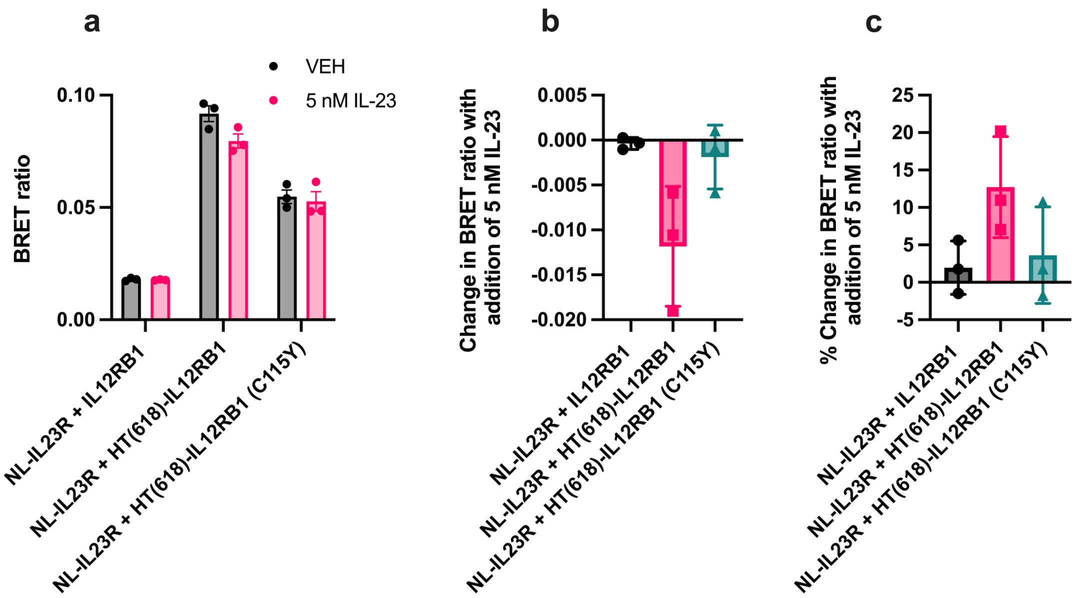


**Supplementary Fig. 3: The C115Y mutant of IL23R associated with IL12Rβ1 but does not undergo ligand induced conformational change.** (a) The BRET ratio generated by transiently transfected cells labelled with HT618 ligand in the presence and absence of IL-23. (b) the change in BRET ratio when the cells from (a) were treated with IL-23. (c) the data from (b) transformed to % change in BRET signal. Data are mean ± SEM from three independent experiments conducted in pentuplicate.

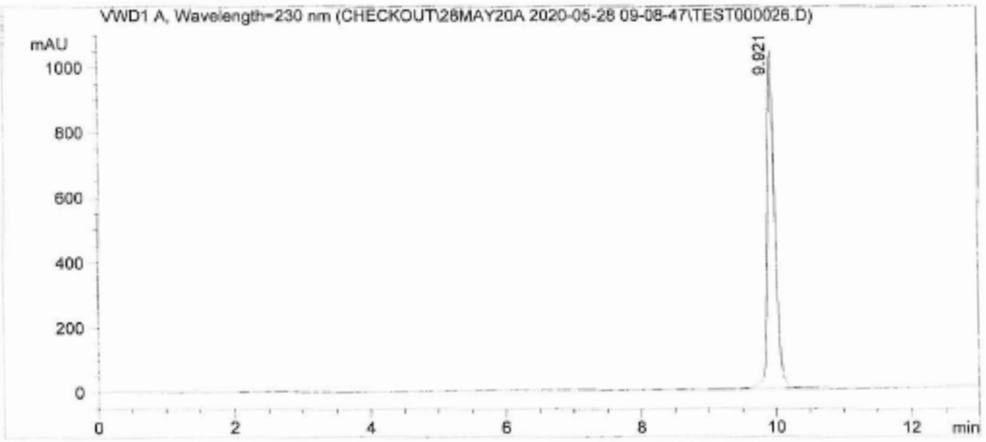


Column: ACE 3 C18-300 300A 150x2.1mm
Buffer A: 0.1 % TFA in H2O
Buffer B: 0.1 % TFA in MeCN
Flow rate: 0.35mL/min
Gradient: 2 to 70% B over 13 min

| # | RT (Min) | Area | Height | Area % |
|---|---|---|---|---|
| 1 | 9.921 | 8081.531 | 1040.918 | 100.0 |

**Supplentary Fig. 4: High Performance Liquid chromatography analysis of P630.** These data were provided by Cambridge Research Biochemicals.

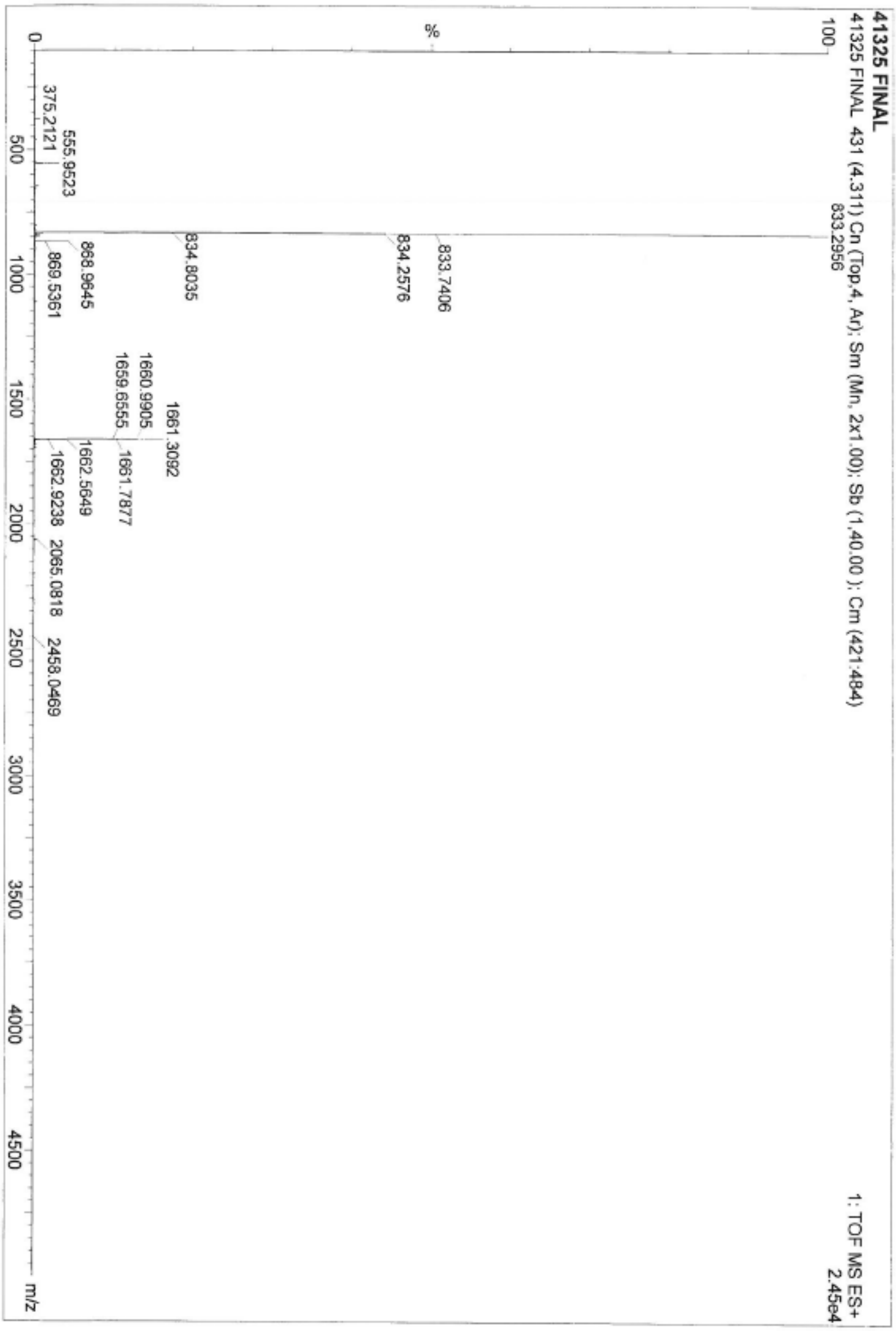


**Supplementary Fig. 5: Mass spectroscopy analysis of P630.** These data were provided by Cambridge Research Biochemicals.

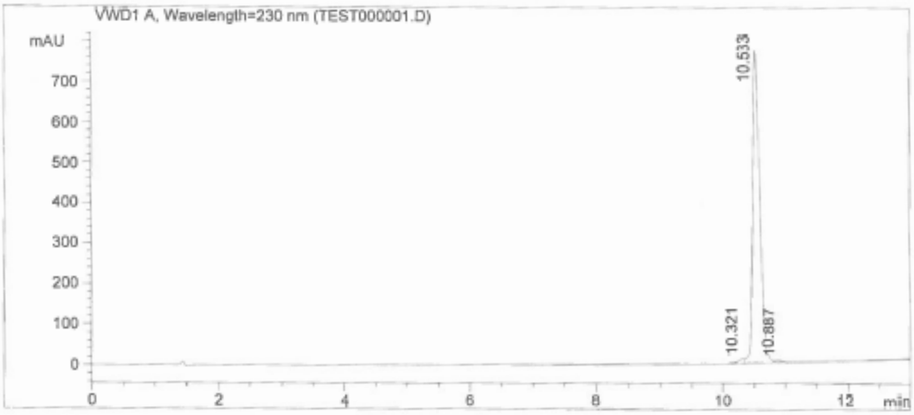


```
Column: ACE 3 C18-300 300A 150x2.1mm
Buffer A: 0.1 % TFA in H2O
Buffer B: 0.1 % TFA in MeCN
Flow rate: 0.35mL/min
Gradient: 2 to 70% B over 13 min
```

| # | RT (Min) | Area | Height | Area % |
|---|---|---|---|---|
| 1 | 10.321 | 63.384 | 9.180 | 1.0 |
| 2 | 10.533 | 6116.216 | 772.829 | 98.4 |
| 3 | 10.887 | 35.843 | 4.312 | 0.5 |

**Supplementary Fig. 6: High Performance Liquid chromatography analysis of P630-TMR.**
These data were provided by Cambridge Research Biochemicals.

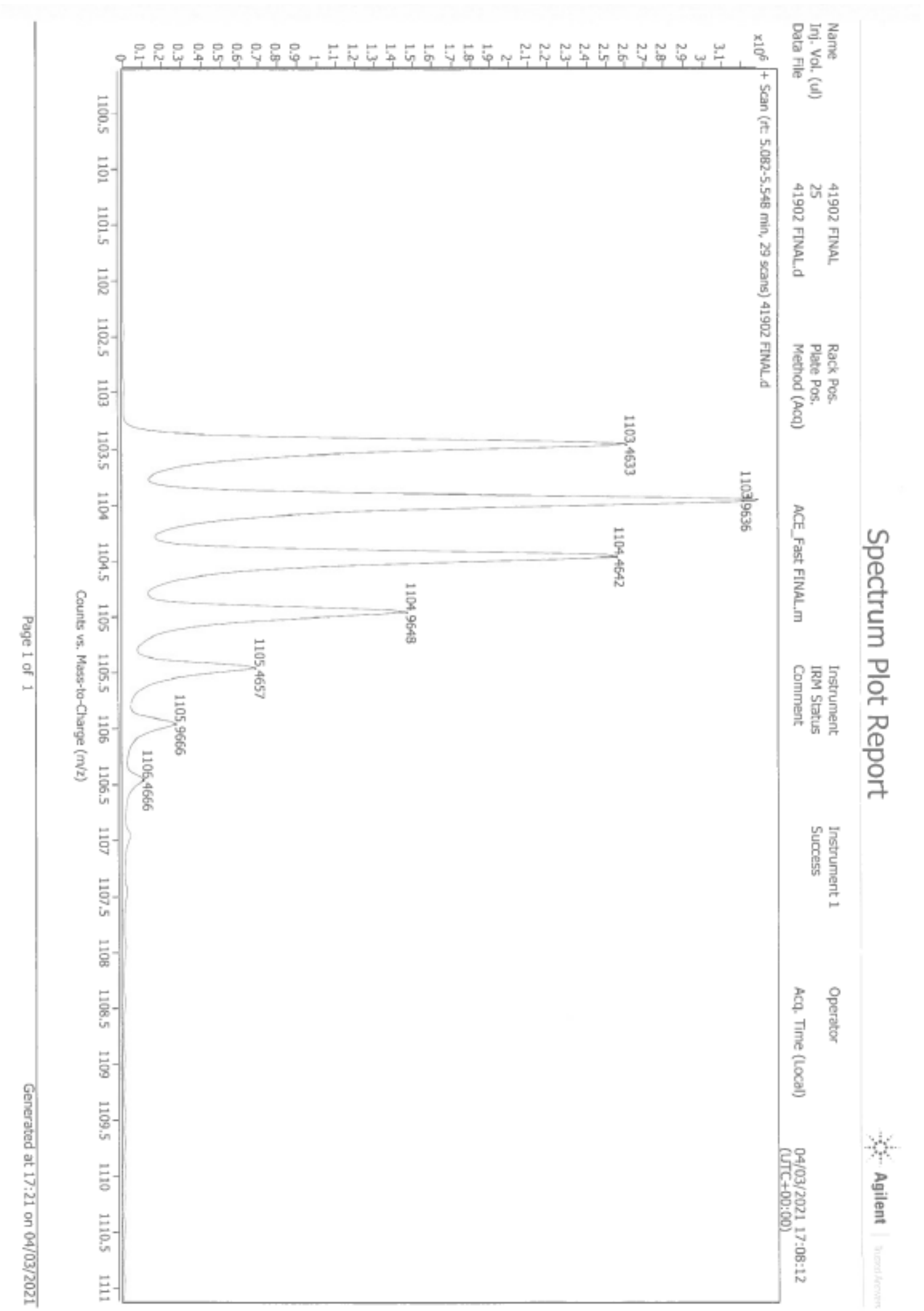


**Supplementary Fig. 7: Mass spectroscopy analysis of P630-TMR.** These data were provided by Cambridge Research Biochemicals.

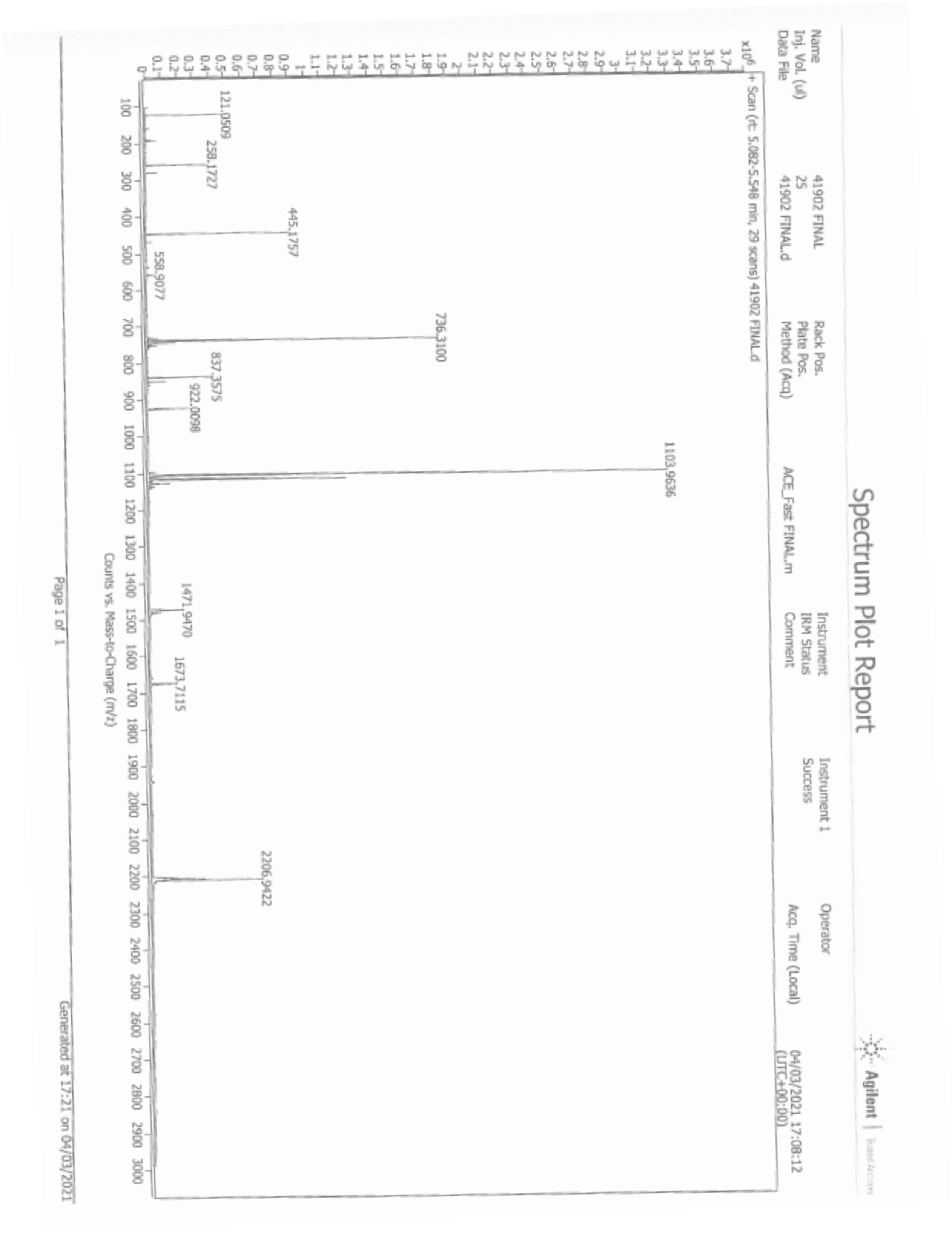


**Supplementary Fig. 8: Mass spectroscopy analysis of P630-TMR.** These data were provided by Cambridge Research Biochemicals.
