# Supplementary material for: Characterisation of IL-23 receptor antagonists and disease relevant mutants using fluorescent probes
Source: Nat Commun. 2023 May 19;14:2882. doi: 10.1038/s41467-023-38541-2 (PMC10199020; doi:10.1038/s41467-023-38541-2)
Supplement: Supplementary file 2 — Reporting Summary [file 41467_2023_38541_MOESM2_ESM.pdf]

nature portfolio

Corresponding author(s): S J Hill

Last updated by author(s): Apr 13, 2023

Reporting Summary

Nature Portfolio wishes to improve the reproducibility of the work that we publish. This form provides structure for consistency and transparency in reporting. For further information on Nature Portfolio policies, see our Editorial Policies and the Editorial Policy Checklist.

Statistics

For all statistical analyses, confirm that the following items are present in the figure legend, table legend, main text, or Methods section.

n/a	Confirmed	
☐	☒	The exact sample size (*n*) for each experimental group/condition, given as a discrete number and unit of measurement
☐	☒	A statement on whether measurements were taken from distinct samples or whether the same sample was measured repeatedly
☐	☒	The statistical test(s) used AND whether they are one- or two-sided<br>*Only common tests should be described solely by name; describe more complex techniques in the Methods section.*
☒	☐	A description of all covariates tested
☐	☒	A description of any assumptions or corrections, such as tests of normality and adjustment for multiple comparisons
☐	☒	A full description of the statistical parameters including central tendency (e.g. means) or other basic estimates (e.g. regression coefficient) AND variation (e.g. standard deviation) or associated estimates of uncertainty (e.g. confidence intervals)
☐	☒	For null hypothesis testing, the test statistic (e.g. *F*, *t*, *r*) with confidence intervals, effect sizes, degrees of freedom and *P* value noted<br>*Give P values as exact values whenever suitable.*
☒	☐	For Bayesian analysis, information on the choice of priors and Markov chain Monte Carlo settings
☒	☐	For hierarchical and complex designs, identification of the appropriate level for tests and full reporting of outcomes
☒	☐	Estimates of effect sizes (e.g. Cohen's *d*, Pearson's *r*), indicating how they were calculated

Our web collection on statistics for biologists contains articles on many of the points above.

Software and code

Policy information about availability of computer code

Data collection	Data were collected using a Pherastar plate reader using the associated BMG labtech data acquisition (version 5.41) and MARS analysis software (version 3.32). Visualisation of
Data analysis	GraphPad Prism (version 9.4.1) was used for data analysis. The crystal structure of N-terminal domains 1-3 of IL23R in complex with IL-23 and the N-terminal domain of IL12Rβ1 (PDB: 6WDQ) was visualised using PyMOL Molecular Graphics System, Version 2.0 Schrödinger, LLC.

For manuscripts utilizing custom algorithms or software that are central to the research but not yet described in published literature, software must be made available to editors and reviewers. We strongly encourage code deposition in a community repository (e.g. GitHub). See the Nature Portfolio guidelines for submitting code & software for further information.

Data

Policy information about availability of data

All manuscripts must include a data availability statement. This statement should provide the following information, where applicable:

- Accession codes, unique identifiers, or web links for publicly available datasets
- A description of any restrictions on data availability
- For clinical datasets or third party data, please ensure that the statement adheres to our policy

The source data for Figures 2-7 and Supplementary Figures 1-3 are provided as a Source Data file. The crystal structure shown in Figure 6a was taken from PDB: 6WDq.

Human research participants

Policy information about studies involving human research participants and Sex and Gender in Research.

Reporting on sex and gender	n/a
Population characteristics	n/a
Recruitment	n/a
Ethics oversight	n/a

Note that full information on the approval of the study protocol must also be provided in the manuscript.

Field-specific reporting

Please select the one below that is the best fit for your research. If you are not sure, read the appropriate sections before making your selection.

☒ Life sciences ☐ Behavioural & social sciences ☐ Ecological, evolutionary & environmental sciences

For a reference copy of the document with all sections, see nature.com/documents/nr-reporting-summary-flat.pdf

Life sciences study design

All studies must disclose on these points even when the disclosure is negative.

Sample size	At least three independent experiments were used to measure each data point. Individual experiments were performed in at least duplicates (technical repeats). Five independent experiments were used for most data sets. Specific numbers of independent experiments are detailed in the figure legends. Sample size was chosen on the basis of the standard deviation obtained in similar plate reader-based experiments.
Data exclusions	Individual data points (individual replicates in an experiment) were excluded on very rare occasions. Data were only excluded from analysis if an experimental error was observed during the set up of the experiment (e.g. pipetting mistake) or a measurement indicated that a particular reagent had not been added (e.g. lack of fluorescence for an added fluorescent probe). One one occasion an outlier was excluded from the statistical analysis in Figure 6b following its identification as an outlier using the Grubbs test (with alpha=0.001). The value of the outlier is, however, given in the legend to Figure 6b.
Replication	Each individual experiment was repeated at least three times. Individual experiments were done in at least duplicates (technical repeats). Five independent experiments were used for most data sets. Specific numbers of independent experiments are detailed in the figure legends and the Source Data file.
Randomization	Experimental conditions were randomly allocated as far as possible within the 96-well design.
Blinding	Blinding of the investigators was not possible for these studies. The transient transfection approach meant that the investigators needed knowledge of the plasmids being used. However, since all data collection was automatic using the PheraStar FS plate reader, observer bias was not considered to be a factor.

Reporting for specific materials, systems and methods

We require information from authors about some types of materials, experimental systems and methods used in many studies. Here, indicate whether each material, system or method listed is relevant to your study. If you are not sure if a list item applies to your research, read the appropriate section before selecting a response.

Materials & experimental systems

n/a	Involved in the study	
☐	☒	Antibodies
☐	☒	Eukaryotic cell lines
☒	☐	Palaeontology and archaeology
☒	☐	Animals and other organisms
☒	☐	Clinical data
☒	☐	Dual use research of concern

Methods

n/a	Involved in the study	
☒	☐	ChIP-seq
☒	☐	Flow cytometry
☒	☐	MRI-based neuroimaging

Antibodies

Antibodies used	Antibodies within the Perkin Elmer Surefire Ultra STAT3 (Tyr705) assay kit (cat number ALSU-PST3) that contains beads coupled to antibodies that recognize STAT3 or phospho-STAT3.
Validation	A positive control lysate is provided as part of the assay kit.

Eukaryotic cell lines

Policy information about cell lines and Sex and Gender in Research

Cell line source(s)	HEK293T (ATCC # CRL-3216)
Authentication	Cells were bought directly from ATCC and were not further authenticated.
Mycoplasma contamination	The cells were tested for mycoplasm and found to be negative.
Commonly misidentified lines (See ICLAC register)	No commonly misidentified cell lines were used in this study. HEK293T cells are not listed in the ICLAC register.
